# Genome-wide identification, expression analysis, and functional study of the GRAS transcription factor family and its response to abiotic stress in sorghum [*Sorghum bicolor* (L.) Moench]

**DOI:** 10.1186/s12864-021-07848-z

**Published:** 2021-07-06

**Authors:** Yu Fan, Jun Yan, Dili Lai, Hao Yang, Guoxing Xue, Ailing He, Tianrong Guo, Long Chen, Xiao-bin Cheng, Da-bing Xiang, Jingjun Ruan, Jianping Cheng

**Affiliations:** 1 College of Agriculture, Guizhou University, Huaxi District, 550025 Guiyang, People’s Republic of China; 2 School of Food and Biological engineering, Chengdu University, 610106 Chengdu, People’s Republic of China; 3 Chengdu Institute of Food Inspection, 610030 Chengdu, People’s Republic of China; 4 Department of Nursing, Sichuan Tianyi College, 618200 Mianzhu, People’s Republic of China; 5 Department of Environmental and Life Sciences, Sichuan MinZu College, 626001 Kangding, People’s Republic of China

**Keywords:** *Sorghum bicolor*, *GRAS* gene family, Genome-wide analysis, Grain development, Abiotic stress

## Abstract

**Background:**

GRAS, an important family of transcription factors, have played pivotal roles in regulating numerous intriguing biological processes in plant development and abiotic stress responses. Since the sequencing of the sorghum genome, a plethora of genetic studies were mainly focused on the genomic information. The indepth identification or genome-wide analysis of *GRAS* family genes, especially in *Sorghum bicolor*, have rarely been studied.

**Results:**

A total of 81 *SbGRAS* genes were identified based on the *S. bicolor* genome. They were named *SbGRAS01* to *SbGRAS81* and grouped into 13 subfamilies (LISCL, DLT, OS19, SCL4/7, PAT1, SHR, SCL3, HAM-1, SCR, DELLA, HAM-2, LAS and OS4). *SbGRAS* genes are not evenly distributed on the chromosomes. According to the results of the gene and motif composition, *SbGRAS* members located in the same group contained analogous intron/exon and motif organizations. We found that the contribution of tandem repeats to the increase in sorghum GRAS members was slightly greater than that of fragment repeats. By quantitative (q) RT-PCR, the expression of 13 *SbGRAS* members in different plant tissues and in plants exposed to six abiotic stresses at the seedling stage were quantified. We further investigated the relationship between DELLA genes, GAs and grain development in *S. bicolor*. The paclobutrazol treatment significantly increased grain weight, and affected the expression levels of all DELLA subfamily genes. *SbGRAS03* is the most sensitive to paclobutrazol treatment, but also has a high response to abiotic stresses.

**Conclusions:**

Collectively, SbGRAs play an important role in plant development and response to abiotic stress. This systematic analysis lays the foundation for further study of the functional characteristics of *GRAS* genes of *S. bicolor*.

**Supplementary Information:**

The online version contains supplementary material available at 10.1186/s12864-021-07848-z.

## Background

Transcription factors (TFs) are a class of proteins that can bind to specific DNA sequences and control the rate of DNA transcription to messenger RNA [1]. This process occurs throughout plant development and regulates complex gene networks in organism, thereby regulating basic aspects of biological functions, including cell differentiation, tissue development, organ construction, metabolic synthesis and environmental adaptation [2].

GRAS is a very important TF family that is unique to plants, named after its three members: GAI (GIBBERELLIC ACID INSENSITIVE) [3], RGA (REPRESSOR OF GA1-3 MUTANT) [4], and SCR (SCARECROW) [5]. In general, the coding sequence (CDS) of *GRAS* TFs is 1200–2100 bp in length, and GRAS proteins are usually between 400 and 700 amino acids long; however, the length and sequence are highly variable [6–9]. Conservation of the GRAS family is reflected in the five highly conserved domains at the C terminus of the protein structure: LHR I (leucine-heptad repeat I), VHIID (Val-His-Ile-Ile-Asp), LHR II, PFYRE (Pro-Phe-Tyr-Arg-Glu), and SAW (Ser-Ala-Trp) [9, 10]. VHIID is considered the core region and it is highly conserved [8–10]; it is located between the two leucine-rich regions LHR I and LHR II, and can combine with them to form an LHR I–VHIID–LHR II complex which plays an important role in DNA and protein binding [11]. It is worth noting that the two leucine-rich regions do not have the 7 repeated leucine residues that form a leucine zipper [7, 12, 13]. The LHR I region has a putative nuclear localization signal near the C terminus, which has been confirmed in the DELLA protein and is similar to the amorphous SV40 (monkey virus) [14]. The latter part of the LHR II domain contains the structure LXXLL (Leu-X-X-Leu-Leu; X represents any amino acid), and it is conserved in over half of the GRAS proteins [15]. The LHR I–VHIID–LHR II domain has been confirmed to be involved in the binding of proteins to nucleic acids and other proteins [16–18]. Although PFYRE is not as strictly conserved as the VHIID region, it still exhibits high similarity in all proteins. Aside from Pro, Phe, Tyr, Arg and Glu, there are also Asp (D) and Glu residues in almost all of the FY domains of GRAS proteins [19]. The SAW region is near the C terminus and usually contains three sequence units: Trp-X7-Gly (WX7G; X7 represents any 7 amino acids), Leu-Trp (LW) and SAW. The SAW unit is present in almost all GRAS proteins. Although the functions of the PFYRE and SAW regions are not known, their high conservation indicates that they are closely associated with GRAS protein functions [19]. The GRAS gene family contains many subfamilies: their protein sequences have great similarities, but also many differences. Early phylogenetic analysis in *Arabidopsis thaliana* divided GRAS proteins into eight subfamilies: DELLA, LS (LATERAL SUPPRESSOR), SCR, SHR (SHORT ROOT), PAT1 (PHYTOCHROME A SIGNAL TRANSDUCTION), HAM (HAIRY MERISTEM), SCL9 (LISCL; Lilium longiflorum SCR-like), and SCL4/7 [20]. Later, Cenci and Rouard [9] proposed that the *GRAS* family members in angiosperms include these eight subfamilies, but also NSP 1, NSP2, DLT (DWARF AND LOW TILLERING), and other subfamilies. Their names are based on the more representative genes in the subfamily, such as DELLA, HAM, DLT, LS, LISCL, PAT1, SCR, SCL3, SHR, and others [6–8].

GRAS proteins also have a variety of functions in the biochemical and physiological processes of plants. *SCR* was the first discovered member of the *GRAS* gene family. It is expressed in roots, leaves and vascular bundle sheath cells with SHR. The *SHR* gene regulates SCR-1 (SCR) and SCR-2 (SCL23), which are involved in the growth of vascular bundle sheath and mesophyll cells in *Arabidopsis* [21]. Both SCR and SHR proteins are involved in regulating the radial growth of *Arabidopsis* roots. As positive regulation factors, they regulate different physiological processes in the formation of radial meristems in roots [5]. *SCL3* has been identified as a target gene of DELLA proteins in *A. thaliana* seedlings [22], and acts as an antagonist of DELLA proteins in controlling the growth of plants by the regulated GA pathway [22, 23]. DELLA proteins not only act as receptors in the gibberellin (GA) reaction [24], but also integrate the signaling pathways of jasmonic acid, auxin, brassinosteroids, and ethylene, constituting a main component of the signaling [25, 26]. The N-terminal IDR of the DELLA subfamily and their protein-binding characteristics extend to all plant-specific GRAS proteins, indicating that the N terminus shifts from disordered to ordered. The transformation is related to the specific binding of GRAS proteins [27]. For example, the protein SLN1 (DELLA) of barley was related to the phosphorylation and dephosphorylation of GA signal-related proteins, initiated by the instability of DELLA inhibitors [28]. In addition, DELLA protein plays an important role in the process of flowering, fruiting and development by inhibiting GAs. On *Lupinus luteus*, *LiDLLA1* was highly involved in early grain development after pollination [29]. The AtPAT 1 subfamily is mainly related to the phytochrome-signaling pathway. For example, PAT1-1 (PAT1), PAT1-2 (SCL21) and PAT1-4 (SCL13) have been found in *Arabidopsis* to be located in the phytochrome-signaling pathway downstream of the transduction pathway [30–32]. Overexpression of *Vitis amurensis* PAT1-1 in *Arabidopsis* can enhance its salt and drought tolerance [33]. LISCL was found in *Lilium longiflorum* to induce meiosis-related promoter lim10 during the microsporogenesis of anthers. LISCL6 (SCL14) was found to play a very important role in activating stress-inducible promoters, especially salicylic acid (SA)- and 2,4-D-inducible promoters, thus participating in the heterologous biochemical processes of detoxifying various substances and harmful endogenous metabolites by regulating its target genes, thereby enhancing plant tolerance to such harmful substances [23]. *SCL* family genes are programmed, during the vegetative growth period of plants, to control the formation of side branches, and they are also present in axillary meristems. For example, overexpression of *Populus euphratica SCL7* in *A. thaliana* enhanced tolerance of the latter to salt and drought stress [34–36]. *HAMII-3* (*SCL6*), *HAMII-2* (*SCL22*) and *HAMII-1* (*SCL27*) in the HAM subfamily inhibit expression of the protochlorophyllide oxidoreductase C (PORC) gene in light-grown plants, negatively regulating plant chlorophyll biosynthesis [37]. *NSPI* and *NSP2* are involved in the synthesis of strigolactones in *Medicago truncatula* and *Oryza sativa*. Strigolactones regulate root branching and attract arbuscular mycorrhizal fungi, and thus have a very important role [38]. DLT protein has been found to reduce grain size in rice [39], and it also has a positive regulatory role in the brassinolide-signaling pathway [40]. Some proteins in the GRAS family play a role through polymerization; for example, the rice DELLA protein SLENDER RICE1 is capable of homodimerization [41].

Sorghum (*Sorghum bicolor*) is a C4 plant with high light use efficiency, easy cultivation, strong adaptability, high nutritional value, which exhibits drought resistance, salt–alkali tolerance and other stress-resistance characteristics. Since the sequencing of the sorghum genome [42], a large number of genetic studies have been carried out. The GRAS is a family of TFs that are unique to higher plants, it plays a vital role in their growth and development, and especially in the morphogenesis of plant roots, hormone signaling, light signaling, and plant stress [4–6]. Most of these factors are characteristic of physiological processes occurring in response to a terrestrial environment. Therefore, the evolution of the *GRAS* gene family provides clues for understanding the adaptive evolution of some C4 plants to environmental changes. The GRAS gene family has been extensively studied in many plant species: once the *GRAS* genes of the model organisms *Arabidopsis* and rice were identified [6–8], these genes could be more widely explored in many other species. This family has been identified and analyzed at the whole-genome level in *Solanum lycopersicum* [43], *Vitis vinifera* [44], castor bean [45], *Malus domestica* [46], *Zea mays* [47], *Camellia sinensis* [48], *Gossypium hirsutum* [49], *Capsicum annuum* [50], *Dendrobium catenatum* [51], *Juglans regia* [52], *Fagopyrum tataricum* [53], *Brassica napu*s [54], *Citrus sinensis* [55], *Hordeum vulgare* [56], *Manihot esculenta* [57], *Lagenaria siceraria*[58] and others. However, knowledge of the *GRAS* gene family in *S. bicolor* is still very limited. The main gene families that have been identified in the sorghum genome are MADS-box [59], Dof [60], CBL [61], ERF [62], SBP-box [63], HSP [64], LEA [65], and NAC [66], among others. Because the *GRAS* genes play important roles in a variety of physiological processes, it is of great significance to systematically study this family in *S. bicolor*. In the present study, we identified 81 *GRAS* genes and divided them into 13 main groups. Their exon–intron structure, motif composition, gene duplication, chromosome distribution and phylogeny were analyzed. The expression of *GRAS* family members in *S. bicolor* during different tissues was also analyzed. Next, we explored the relationship between DELLA and *S. bicolor* grain development. Finally, the expression of *GRAS* family members under six abiotic stresses were also analyzed. These data provide useful information for the study of the evolutionary relationship and biological function of the *SbGRAS* gene family.

## Results

### Identification of *GRAS* genes in *S. bicolor*

In this study, we used two BLAST methods to identify all possible *GRAS* members in the *S. bicolor* genome. Ultimately, 81 *SbGRAS* genes were identified (Additional file 1: Table S1). They were named *SbGRAS01* to *SbGRAS81* according to their chromosomal location. The basic characteristics were analyzed, including CDS length, protein molecular mass, isoelectric point (pI), domain information and subcellular localization (http://cello.life. nctu.edu.tw/) (Additional file 1: Table S1). Of the 81 SbGRAS proteins, SbGRAS80 was the smallest with 174 amino acids, and the largest was SbGRAS35 with 968 amino acids. The molecular masses of the proteins ranged from18.99 kDa (SbGRAS80) to 107.47 kDa (SbGRAS35), and the pI ranged from 4.82 (SbGRAS66) to 9.05 (SbGRAS30), with a mean of 6.01. The CDS lengths of the *SbGRAS* genes varied greatly, from 522 bp (*SbGRAS*80) to 2904 bp (*SbGRAS35*). The predicted subcellular localization results showed that 37 SbGRAS proteins were located in the nuclear region, 24 in the cytoplasm, and 20 in the chloroplast (Additional file 1: Table S1).The number of GRAS TFs in *S. bicolor* exceeded that in *A. thaliana* (32) and rice (57) [8], *Cucumis sativus* (37) [67], *Vitis vinifera* (52) [44] and Tartary buckwheat (47) [53], whereas there were fewer TFs than that in *Malus* x *domestica* (127) [46] and *Populus trichocarpa* (102) [68]. The ratio of *SbGRAS* genes to total number of genes in the *S. bicolor* genome was about 0.27 %, which is more than in *Arabidopsis* (0.11 %) [6], rice (0.15 %) [8], tomato (0.15 %) [8], *Cucumis sativus* (0.14 %) [67] and Tartary buckwheat (0.14 %) [53], but less than in *Carica papaya* (0.31 %) [68] and *Medicago truncatula* (0.29 %) [69]. Previous studies have shown that the number and density of GRAS proteins are related to genome size and repetitive events. Therefore, some important GRAS proteins are retained during genome replication to adapt to complex environments [68].

### Multiple sequence alignment, phylogenetic analysis, and classification of SbGRASs

**Fig. 1:**
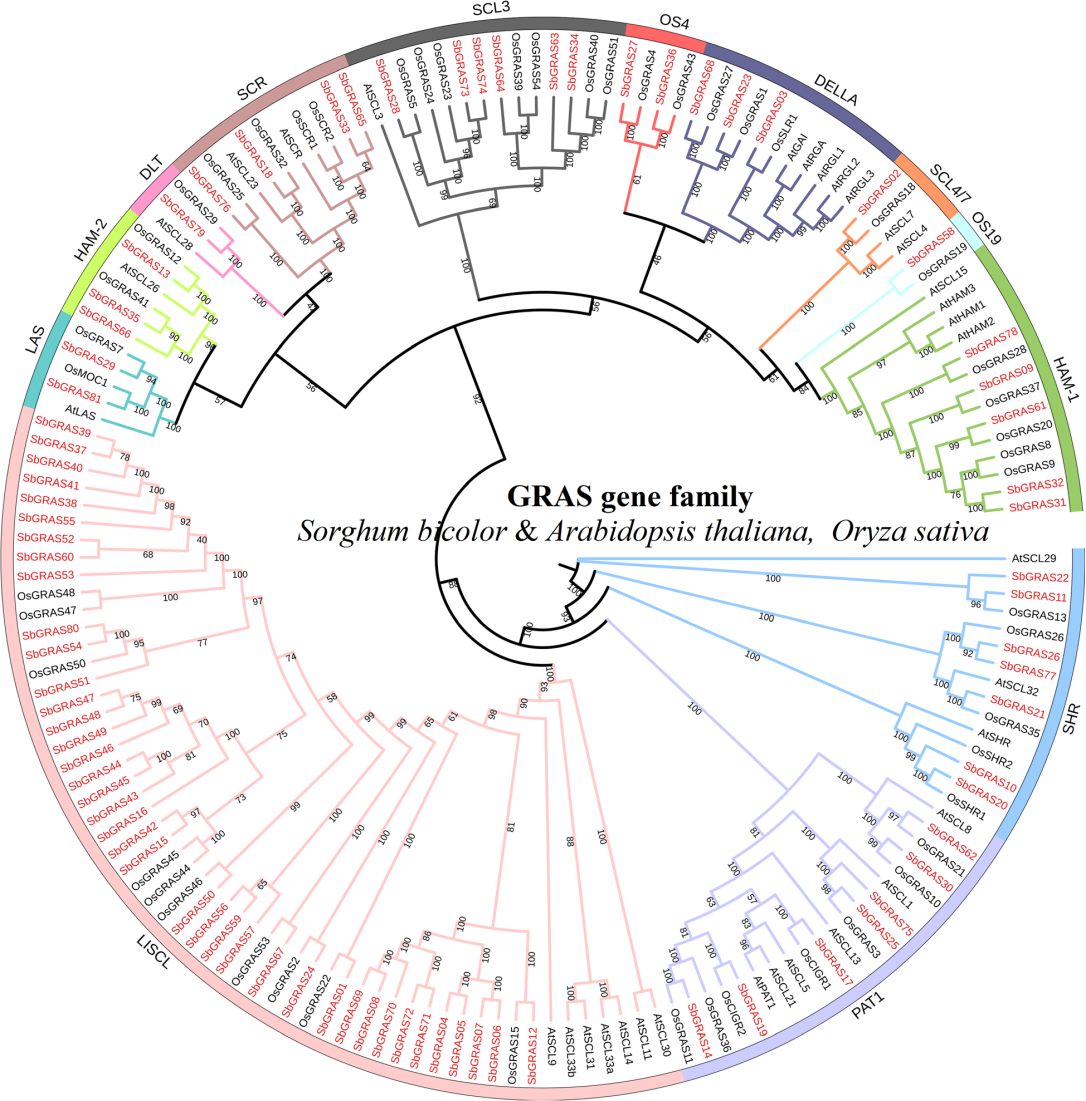
Unrooted phylogenetic tree representing relationships among GRAS domains of *S. bicolor*, *Arabidopsis* and rice. The phylogenetic trees were derived using the NJ method in MEGA7.0. The tree shows the 13 phylogenetic subfamilies marked with red font on a white background. GRAS proteins from *Arabidopsis* and *Oryza sativa* have the prefix ‘At’ and ‘Os’, respectively

We constructed a phylogenetic tree using the neighbor-joining (NJ) method with a bootstrap value of 1000 based on the amino acid sequences of the 81 identified SbGRAS, 33 AtGRAS and 50 OsGRAS proteins (Fig. 1, Additional files 1 and 2: Tables S1 and S2). According to the topological structure of the tree and the classification method proposed by Cenci and Rouard [9], the 164 GRAS proteins in the phylogenetic tree were divided into 13 clades (groups 1–13), consistent with the tree topology and previous classification of the GRAS taxonomic group in angiosperms [9], and indicating no loss of those proteins during *S. bicolor*’s evolution. These findings revealed that GRAS proteins within the reported subfamilies that are present in different plant species play a basic role in plant development and evolution, similar to those recently reported in previous studies on some other plant species, including *Amborella trichopoda*, *Phoenix dactylifera*, *Vitis vinifera*, *Musa acuminata*, *O. sativa*, *A. thaliana*, *Theobroma cacao* and *Coffea canephora* [9]. Among the 13 subfamilies, LISCL had the most members (39 SbGRASs), and DLT (*SbGRAS79*), OS19 (*SbGRAS58*) and SCL4/7 (*SbGRAS02*) had the fewest (1 *SbGRAS*). There were 7, 7, 6, 5, 4, 3, 3, 2 and 2 *SbGRAS* genes in the PAT1, SHR, SCL3, HAM-1, SCR, DELLA, HAM-2, LAS and OS4 groups, respectively (Fig. 1, Additional file 1: Table S1). The phylogenetic tree with *A. thaliana* and *O. sativa* showed that some SbGRASs were tightly grouped with the OsGRASs (bootstrap support ≥ 70). These results indicate that the GRAS proteins may have evolved further after the separation of monocotyledons and dicotyledons in angiosperms. These proteins may be orthologous to the OsGRASs and have similar functions.

**Fig. 2:**
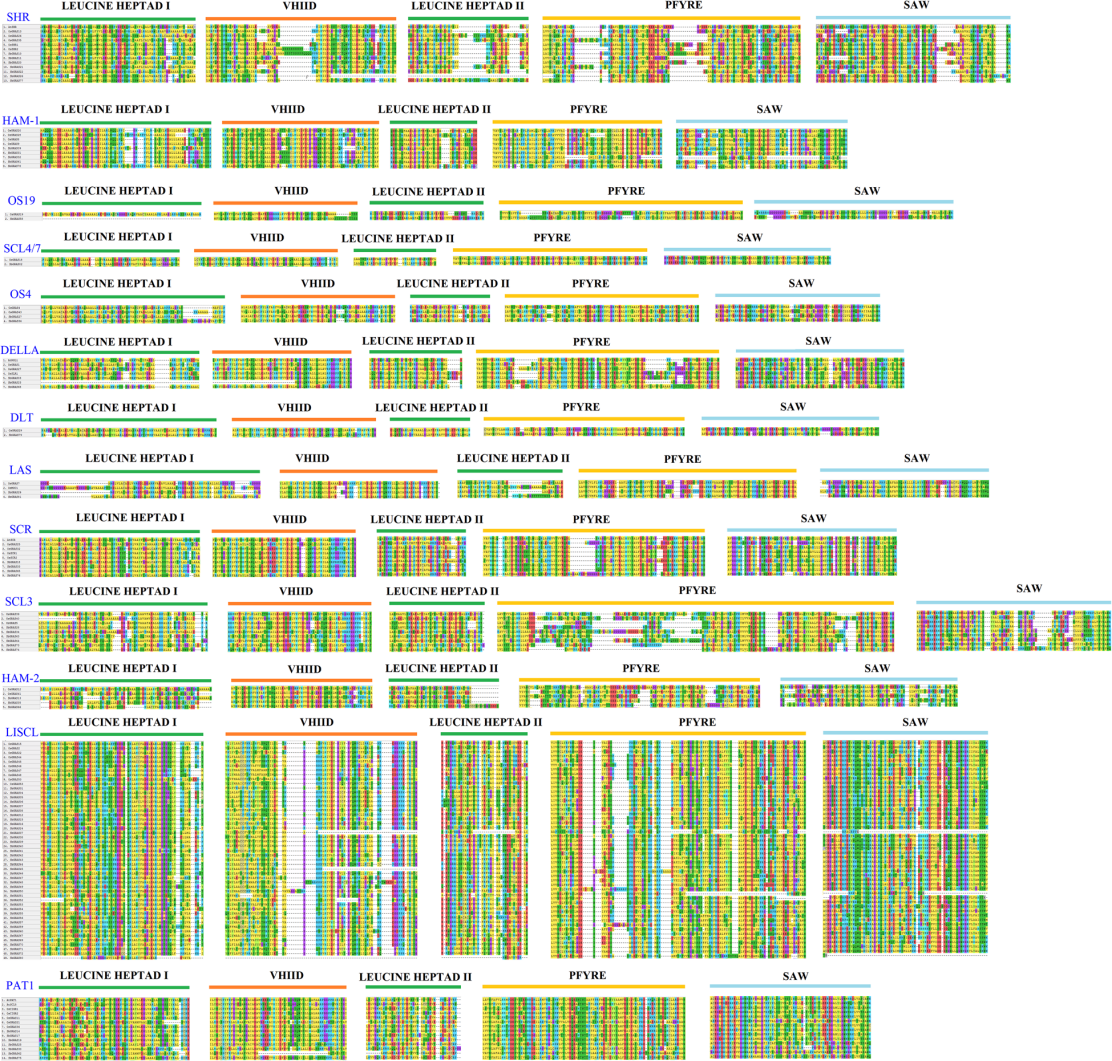
Multiple sequence alignment of the GRAS domains of the members of 13 phylogenetic subfamilies of the *S. bicolor* GRAS protein family. The scheme at the top depicts the locations and boundaries of the LHR I, VHIID, LHR II, PFYRE, and SAW regions within the GRAS domain

The GRAS proteins of *Arabidopsis* and rice were randomly selected and their LHR I, VHIID, LHR II, PFYRE, and SAW domains were further compared. As shown in Fig. 2, the VHIID domain contains a characteristic amino acid sequence, and is considered to be the core region. Although its structure in the different species was highly similar and easy to identify, it was not absolutely conserved. The His and Asp residues in the domain were more conserved. It is worth noting that we divided HAM into groups HAM-1 and HAM-2 based on their genetic and developmental relationships and the results of multiple sequence alignments. Compared to HAM-1, HAM-2 has a more conserved N terminus and VHIID region; it had a smaller number of amino acids in SbGRAS13, SbGRAS35, and SbGRAS66. Among the 81 identified SbGRAS proteins, we observed some domain-loss events in SbGRAS23, SbGRAS26, SbGRAS58, and SbGRAS62, a phenomenon that often occurs in monocots [70].

### Conserved motifs and gene structure analysis of *SbGRAS* genes

**Fig. 3:**
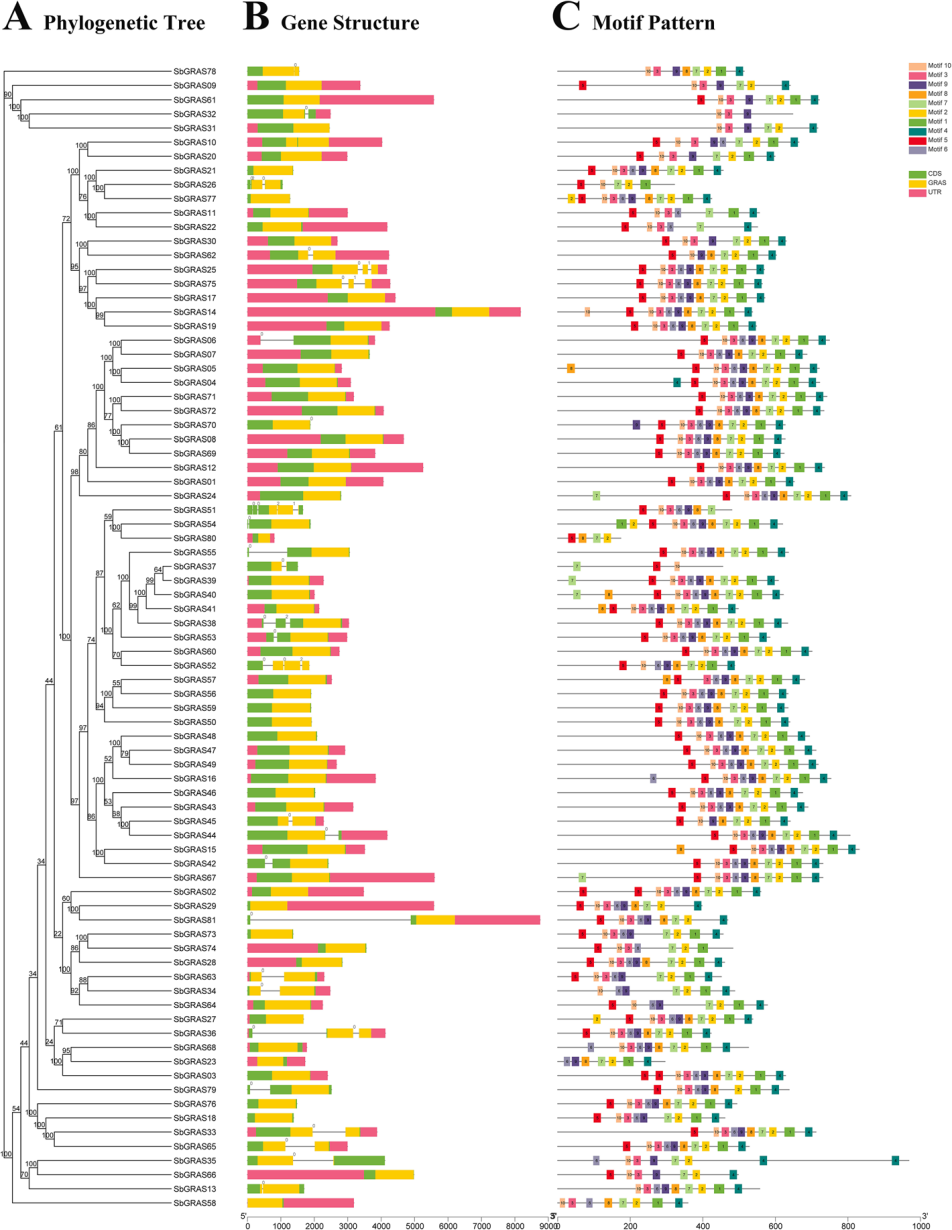
Phylogenetic relationship, gene-structure analysis, and motif distributions of *S. bicolor GRAS* genes. **A** Phylogenetic tree was constructed by the NJ method with 1000 replicates on each node. **B** Exons and introns are indicated by green rectangles and gray lines, respectively. These numbers are generated based on “phase” of the annotate file, which is about the different phases of CDS in gene. Meanwhile, “phase” is defined as “0”, “1”, and “2”. **C** Amino acid motifs in the *S. bicolor* GRAS proteins (1–10) are represented by colored boxes. The black lines indicate relative protein lengths

To understand the structural components of the *SbGRAS* genes, their exon and intron structures were obtained by comparing the corresponding genomic DNA sequences (Fig. 3, Additional files 1 and 3: Tables S1 and S3). By comparing the number and position of the exons and introns, we found that the 81 identified *SbGRAS* genes have different numbers of exons, varying from 1 to 5 (Fig. 3A/B). The 81 *SbGRAS* genes all contained the GRAS domain, and most of the *SbGRAS* genes (54, ~ 67.5 %) contained no introns; 19 *SbGRAS* genes contained 1 intron; *SbGRAS25*, *SbGRAS36*, *SbGRAS38* and *SbGRAS75* contained 2 introns; *SbGRAS52* and *SbGRAS26* contained 3 introns. *SbGRAS51* had the most introns: 4. The 54 intron-less genes were distributed across the other 12 subfamilies, except for the DLT subfamily, and mainly in the LISCL subfamily. In general, members of the same subfamily had similar gene structures. Members of the groups DELLA, OS19, SCL4/7, DLT, HAM-1, HAM-2, LAS, SCL3 and SCR contained 0 or 1 intron. Further analyses indicated that the LISCL group was most diverse in terms of number of introns.

To further study the characteristic regions of the SbGRAS proteins, their motifs were analyzed using an online MEME. A total of 10 distinct conserved motifs (named motifs 1–10) were found (Fig. 3C, Additional file 3: Table S3). As exhibited in Fig. 3C, motif 10 was widely distributed in the SbGRASs, except for SbGRAS23, SbGRAS57 and SbGRAS80, and was always close to motifs 5 and 3. SbGRAS members of the same group usually shared a similar motif composition. For example, group SCL3 contained motifs 10, 6, 7, 2; group PAT1 contained motifs 5, 10, 9, 7, 2, 1, 4; group SHR contained motifs 5, 10, 7, 1. Some motifs were only distributed in specific locations of the pattern. For example, motifs 5 and 10 were always distributed at the start of the pattern, and motif 4 was almost always at the end of the pattern. The functions of most of these conserved motifs remain to be elucidated.

### Chromosomal spread and gene duplication in *SbGRAS* genes

**Fig. 4:**
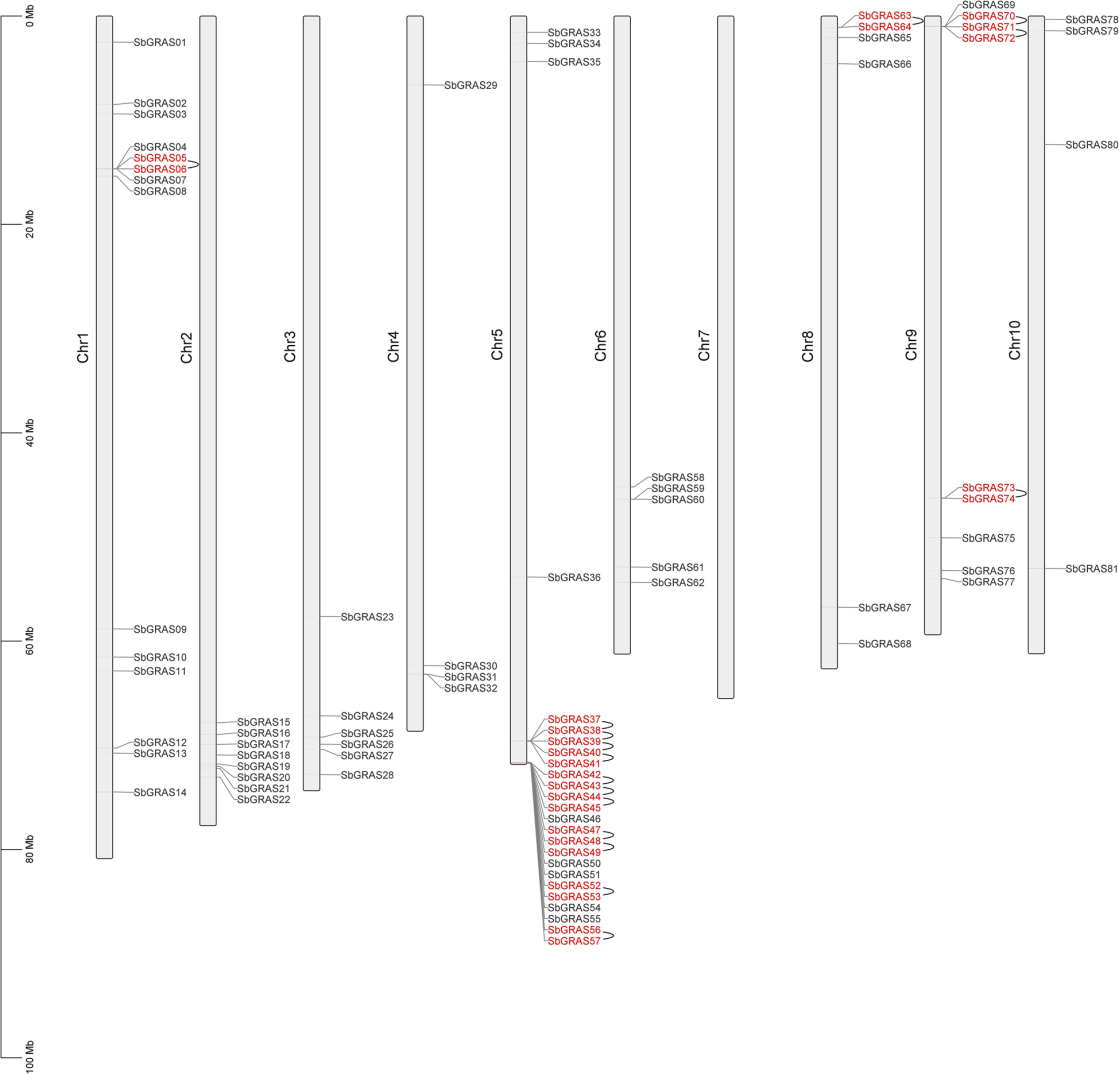
Schematic representations of the chromosomal distribution of the *S. bicolor GRAS* genes. Vertical bars represent the chromosomes of *S. bicolor*. The chromosome number is indicated to the left of each chromosome. The scale on the left represents chromosome length

A map of the physical position of the *SbGRAS* genes was created based on the *S. bicolor* genome database (Fig. 4, Additional files S1 and S4: Tables S1 and S4). Each *SbGRAS* was named according to its physical position from the top to the bottom of *S. bicolor* chromosomes (Chr) 1 to 10. The distribution of the 81 *SbGRAS* genes on the chromosomes was uneven. Interestingly, *SbGRAS* genes were not found on Chr7. We speculate that this is due to fragment loss or chromosome shift during evolution. Chr5 contained the largest number of *SbGRAS* genes (25 genes, ~ 30.86 %), followed by Chr1 (14, ~ 17.28 %); Chr4 and Chr10 contained the least *SbGRAS* genes (4 each, ~ 4.94 %). Chr9 and Chr8 contained 9 (~ 11.11 %) and 8 (~ 9.88 %) *SbGRAS* genes, respectively. Chr3, Chr6, and Chr8 contained 6 (~ 7.41 %), 5 (~ 6.17 %), and 6 (~ 7.41 %) *SbGRAS* genes, respectively. A chromosomal region within 200 kb containing 2 or more genes is defined as a tandem duplication event [71]. On Chr1, 5, 8 and 9, we found 16 tandem duplication events involving 25 *SbGRAS* genes (Fig. 4). *SbGRAS38*, *SbGRAS39*, *SbGRAS40*, *SbGRAS43*, *SbGRAS44*, *SbGRAS48* and *SbGRAS71* each had 2 tandem repeat events (*SbGRAS38* and *SbGRAS37*/*SbGRAS39*; *SbGRAS39* and *SbGRAS38*/*SbGRAS40*; *SbGRAS40* and *SbGRAS39*/*SbGRAS41*; *SbGRAS43* and *SbGRAS42*/*SbGRAS44*; *SbGRAS44* and *SbGRAS43*/*SbGRAS45*; *SbGRAS48* and *SbGRAS47*/*SbGRAS49*; *SbGRAS71* and *SbGRAS70*/*SbGRAS72*). All of the genes that showed tandem repeat events were members of the same subfamily. It was further discovered that 14 of the 16 pairs were from subfamily LISCL, indicating its important evolutionary role in gene expansion, and indeed, it was the largest subfamily. Only *SbGRAS63*/*SbGRAS64* and *SbGRAS72*/*SbGRAS73* were from subfamily SCL3.

**Fig. 5:**
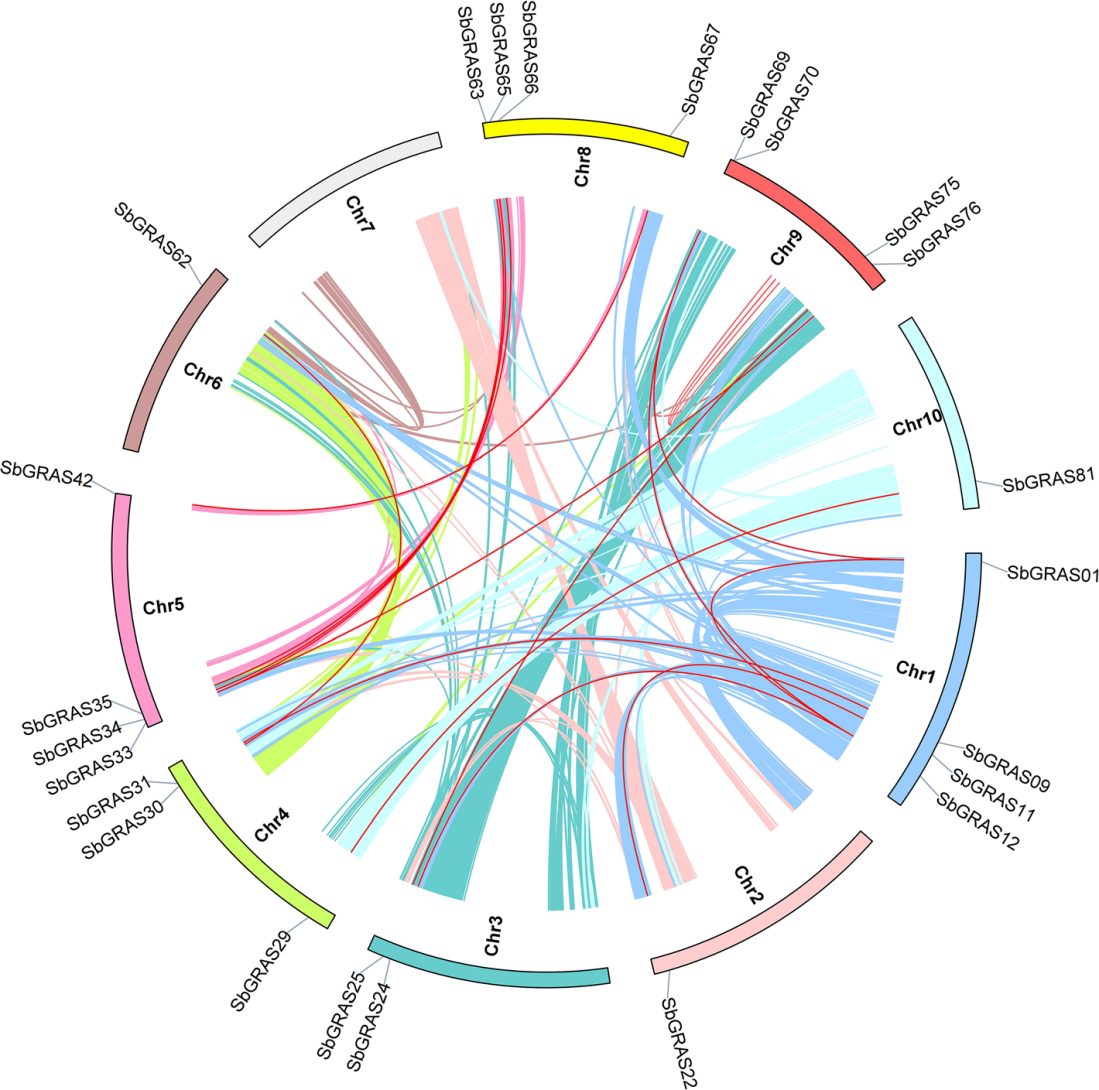
Schematic representations of the chromosomal distribution and segmental duplication relationships of *S. bicolor GRAS* genes. Colored lines indicate all synteny blocks in the *S. bicolor* genome and the red lines indicate duplicated *GRAS* gene pairs. The chromosome number is indicated at the bottom of each chromosome

In addition, there were 14 pairs of segmental duplications in the *SbGRAS* genes (Fig. 5, Additional file 5: Table S5). As shown in Fig. 5, 24 (~ 29.63 %) paralogs were identified in the *SbGRAS* gene family, indicating an evolutionary relationship for these *GRAS* members. The *SbGRAS* genes were unevenly distributed in 10 *S. bicolor* linkage groups (LG). Some linkage groups had more *SbGRAS* genes than others, for example, LG1, LG5, LG8 and LG9 had 4 *SbGRAS* genes, whereas LG2, LG6 and LG10 had only 1 *SbGRAS* gene. Further analysis of these genes’ subfamilies showed that all of them were linked within their subfamily. For example, *SbGRAS33* and *SbGRAS65/76* were segmental duplications and they clustered together (subfamily SCR) (Fig. 5, Additional file 5: Table S5). Out of all identified *SbGRAS* genes, group LISCL had the largest number of linked genes (7/24, ~ 29.17 %). In addition, group PAT1 had 4 segmental duplications, while groups HAM-1, HAM-2, LAS, SCL3, SHR has only one pair of segmental duplications ( Additional file 5: Table S5).

### Synteny analysis of *SbGRAS* genes

**Fig. 6:**
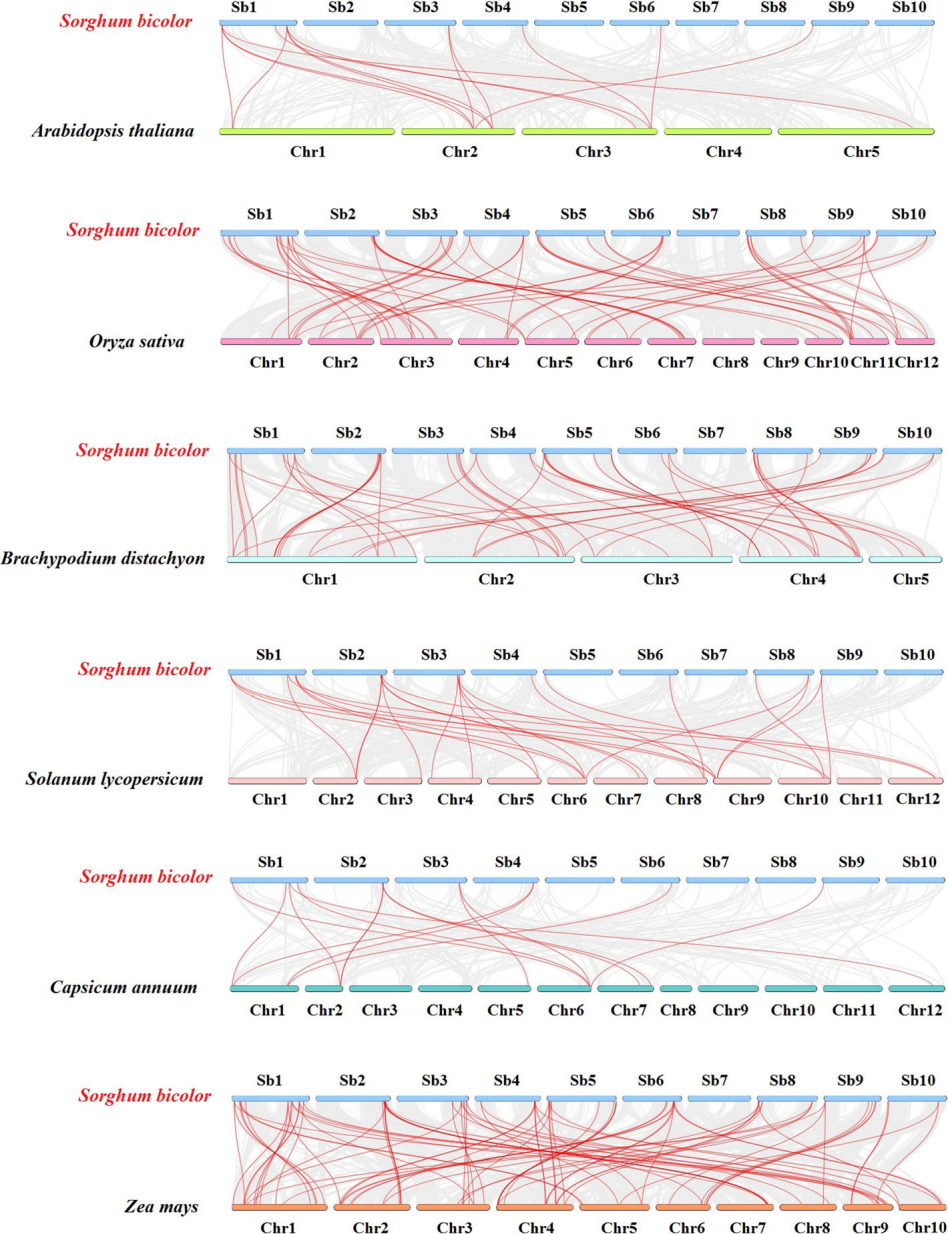
Synteny analyses of the *GRAS* genes between *S. bicolor* and six representative plant species. Gray lines on the background indicate the collinear blocks within *S. bicolor* and other plant genomes; red lines highlight the syntenic *S. bicolor GRAS* gene pairs

To further infer the phylogenetic mechanisms in the *SbGRAS* family, we constructed six comparative syntenic maps of *S. bicolor* with six representative species: three dicotyledons (*A. thaliana*, *C. annuum* and *Solanum lycopersicum*) and three monocotyledons (*O. sativa*, *Brachypodium distachyon* and *Z. mays*) (Fig. 6, Additional file 6: Table S6). A total of 51 *SbGRAS* genes showed syntenic relationships with those in *Arabidopsis* (6), followed by *C. annuum* (9), tomato (14), *B. distachyon* (37), indica rice (39) and maize (65) (Additional file 6: Table S6). The number of orthologous pairs between the other six species (*Arabidopsis*, *C. annuum*, tomato, *B. distachyon*, indica rice and maize) was 14, 15, 27, 51, 56 and 90, respectively.

Some *SbGRAS* genes were found to be associated with at least one syntenic gene pair among the six plants (especially between *S. bicolor* and *Z. mays GRAS* genes), such as *SbGRAS01*, *SbGRAS12*, *SbGRAS24*, *SbGRAS31*, suggesting that these orthologous pairs already existed before the ancestral divergence, and thus indicating that these genes may have played an important role in the *GRAS* gene family during evolution. Interestingly, some collinear gene pairs (with 12 *SbGRAS* genes) identified between *S. bicolor* and *B. distachyon*/indica rice/maize were not found between *S. bicolor* and *Arabidopsis*/*C. annuum*/tomato, such as *SbGRAS02*, *SbGRAS03*, *SbGRAS18*, *SbGRAS23*, *SbGRAS29*, *SbGRAS30*, *SbGRAS34*, *SbGRAS35*, *SbGRAS36* and *SbGRAS42*. This suggests that these orthologous pairs may be formed after the divergence of dicotyledonous and monocotyledonous plants (Additional file 6: Table S6). Significantly, some collinear *GRAS* gene pairs identified between *S. bicolor* and indica rice/maize/*B. distachyon* were anchored to highly conserved syntenic blocks that spanned 18 genes. In contrast, those between *S. bicolor* and *Arabidopsis*/*C. annuum*/tomato were all located in syntenic blocks that had less than 10 orthologous gene pairs. This might be related to the phylogenetic relationship between *S. bicolor* and the other six plant species.

To better understand the evolutionary constraints acting on the *SbGRAS* gene family, the *SbGRAS*s were subjected to Tajima’s D Neutrality Test [72, 73]. Calculations gave D = 7.25577; the large deviation from 0 suggests that the *SbGRAS* gene family might have experienced strong purifying selective pressure during evolution (Additional file 7: Table S7). Positive selection was analyzed between sequences using MEGA 7.0 [74, 75]. The results showed that some *SbGRAS* members were relatively more favored by Darwinian selection, which is the evidence that some *SbGRAS* proteins are more adaptive to evolution (Additional file 8: Table S8).

### Evolutionary analysis of the *SbGRAS* genes and *GRAS* genes of several different species

**Fig. 7:**
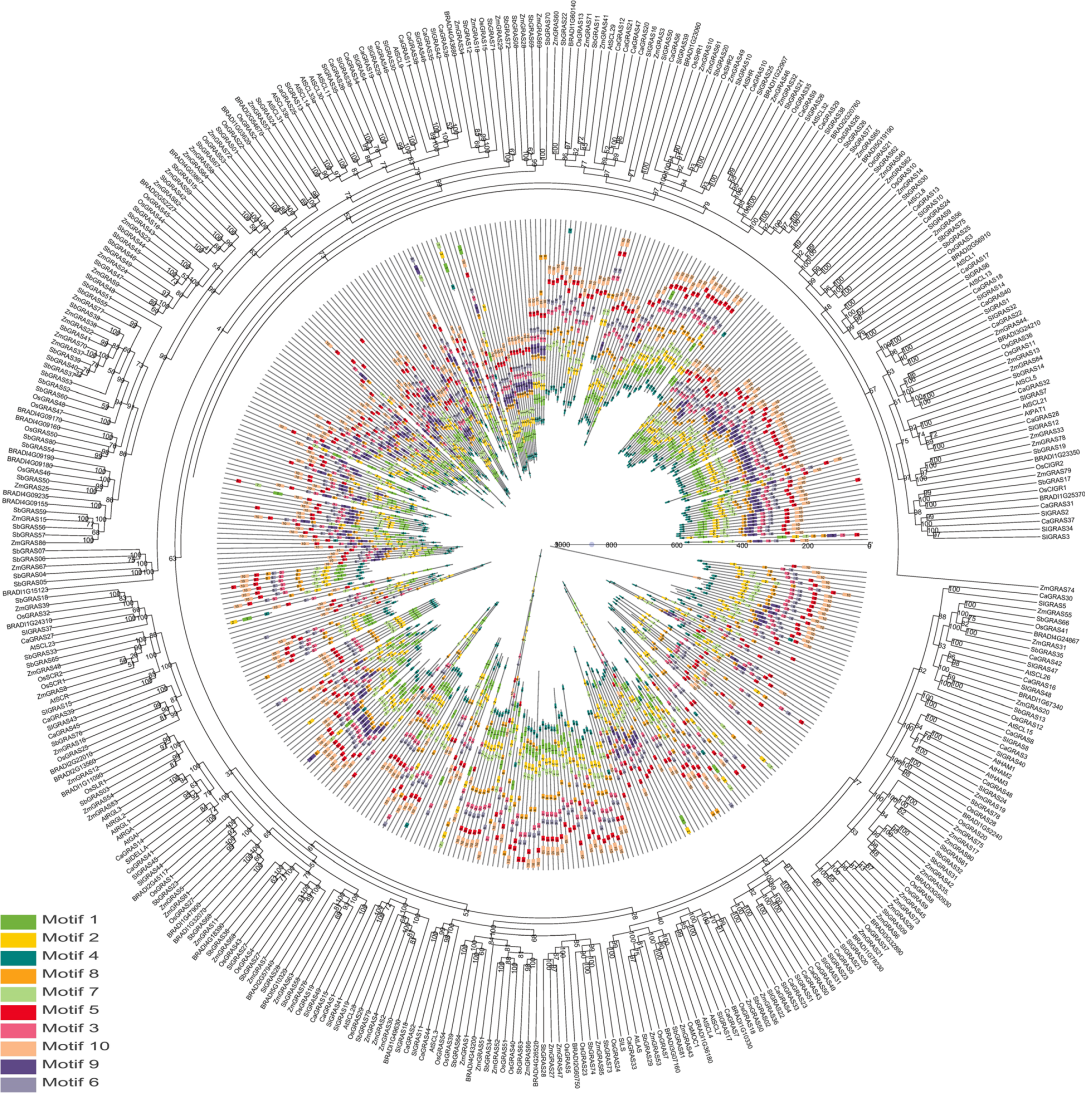
Phylogenetic relationships and motif compositions of the *S. bicolor* GRAS proteins with six different plant species (*Arabidopsis thaliana*, *Capsicum annuum*, *Solanum lycopersicum*, *Brachypodium distachyon*, *Oryza sativa* subsp. *indica*, *Zea mays*). Outer panel: Unrooted phylogenetic tree constructed using Geneious R11 with the NJ method. Innermost panel: Distribution of the conserved motifs in GRAS proteins. The differently colored boxes represent different motifs and their positions in each GRAS protein sequence. The sequence information for each motif is provided in Additional file 3: Table S3

To analyze the evolutionary relations between the trihelix family of *SbGRAS* proteins among *S. bicolor* and six plants (*A. thaliana*, *C. annuum*, *Solanum lycopersicum*, *B. distachyon*, *O. sativa* subsp. *indica*, *Z. mays*), an unrooted NJ tree with 10 conserved motifs according to the MEME web server was constructed using the NJ method of Geneious R11 according to the protein sequences of the 81 identified *SbGRAS* genes and the six other plants’ trihelix genes (Fig. 7, Additional file 3: Table S3). The distribution of *SbGRAS*s in the phylogenetic tree was relatively widely dispersed. As shown in Fig. 7, the SbGRAS proteins tended to gather with the GRAS proteins of *O. sativa* and *Z. mays*, suggesting that they are more closely related. Most of the GRAS proteins from the six studied plants contained motifs 5, 10 and 2. In addition, several motifs were only present in the GRAS proteins of a few specific *SbGRAS* branches, such as motif 1. Motif 5 was distributed between motifs 10 and 6, and motif 7 was distributed between motifs 2 and 8. Motif 10 was always distributed at the start of the pattern and motif 4 was almost always distributed at the end of the pattern. We also found that the GRAS proteins of *O. sativa*, *Z. mays* and *S. bicolor* on the same branch generally had similar motif compositions, and similar serial motifs tended to cluster in specific GRAS protein subfamilies, indicating potential functional similarities between those GRAS proteins.

### Expression patterns of *SbGRASs* in several organs

**Fig. 8:**
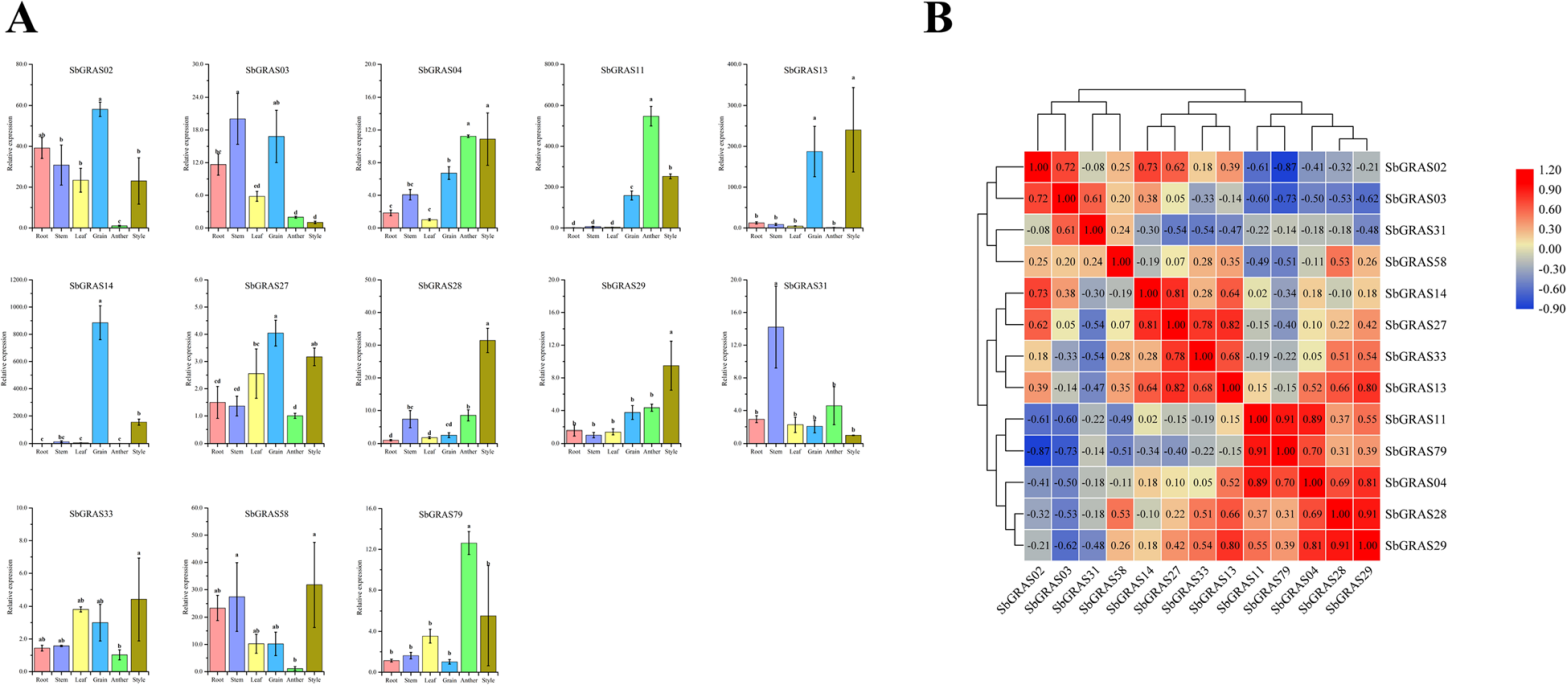
Tissue-specific expression of 13 *S. bicolor GRAS* genes and the correlation between their expression patterns. **A** Expression patterns of 13 *S. bicolor GRAS* genes in anther, style, leaf, root, stem and grain organs were examined by qRT-PCR. Error bars were obtained from three measurements. Lowercase letters above the bars indicate significant differences (α = 0.05, LSD) among the treatments. The SE is selected as the value of bar. The same below. **B** Positive number: positively correlated; negative number: negatively correlated. Red numbers indicate a significant correlation at the 0.05 level

To further analyze the potential roles of *SbGRAS* genes, we randomly selected 1 gene in each subfamily and tested the expression of these 13 representative genes in six organs (anthers, styles, grain, roots, stems, leaves) by qRT-PCR assay (Fig. 8A). The expression patterns of the *SbGRAS* genes changed substantially among the six plant organs, with some exhibiting preferential expression across the detected tissues of *S. bicolor*. Most of genes were expressed in all organs; 2 genes (*SbGRAS11* and *SbGRAS79*) showed the highest expression level in the anther; *SbGRAS28* and *SbGRAS29* showed the highest expression level in the style, and the highest expression of *SbGRAS02*, *SbGRAS14* and *SbGRAS27* was found in the grain; 2 genes (*SbGRAS03* and *SbGRAS31*) were highly expressed in the stem. The results showed diverse transcriptional abundance of *SbGRAS* genes in different tissues and organs, indicating that these genes have multiple functions in sorghum growth and development of. For example, the expression level of *SbGRAS03* of the DELLA family was high during the grain-filling stage, suggesting an important role in sorghum grain development. Correlations of *SbGRAS* gene expression among the six organs were also studied (Fig. 8B). We found that the expression of different genes in the plant organs was significantly correlated, indicating their possible synergistic role. Most *SbGRAS* genes showed significant positive correlations; for example, *SbGRAS04*, *SbGRAS11*, *SbGRAS28*, *SbGRAS29*, and *SbGRAS79* were all highly expressed in the style and anther, and their expression was significantly positively correlated. However, these same genes were significantly negatively correlated with *SbGRAS02*, *SbGRAS03*, and *SbGRAS31*.

### Effects of grain development and expression of DELLA subfamily genes after Paclobutrazol treatment

**Fig. 9:**
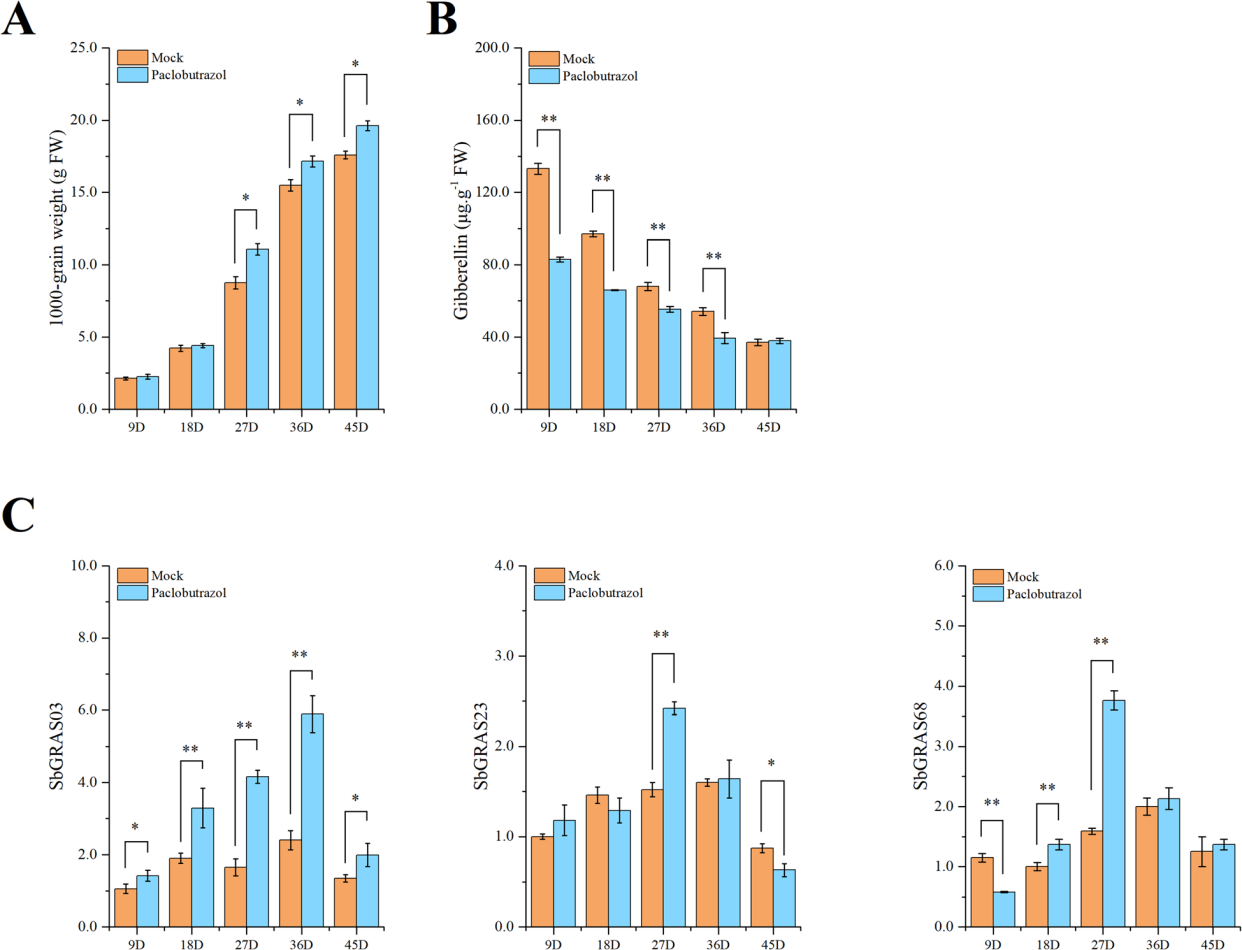
Grain development of S. bicolor under exogenous paclobutrazol treatment. **A** 1000 grain weight during grain development. **B** Gibberellin content during grain development. **C** Differences in the expression of DELLA subfamily genes under exogenous paclobutrazol treatment during grain development. Mock: the same amount of water treatment, paclobutrazol: 750 mg·L − 1 paclobutrazol treatment. Error bars were obtained from three measurements. Small letter(s) above the bars indicate significant differences (α = 0.05/0.01, LSD) among the treatments. * and ** indicate significant correlations at the 0.05 and 0.01 levels, respectively

Paclobutrazol is a highly effective and low toxicity plant growth regulator, which can delay plant growth and regulate grain development [76, 77]. In order to further investigate the relationship between paclobutrazol and the regulation of grain development on *S. bicolor*, the 1000 grain weight and GA content at different stages of grain development after paclobutrazol and blank treatment (Mock) were observed (Fig. 9A/B). The results showed that the 1000-grain weight of sorghum was increased by paclobutrazol treatment, particularly in the later stage of grain development. Additionally, the GAs of both Mock and paclobutrazol treatment groups decreased during grain development, and the paclobutrazol treatment group dropped to a lower level more dramatically (37.88 µg·g^− 1^). DELLA protein is a negative regulator in the pathway of GAs metabolism, which can regulate grain development by responding to external signals [78]. In order to figure out the important regulation effects of DELLA subfamily proteins in the grain development of *S. bicolor*, the expression of DELLA subfamily genes (*SbGRAS03*, *SbGRAS23*, *SbGRAS68*) with 750 mg·L^− 1^ exogenous paclobutrazol was further compared (Fig. 9C). The experimental and control groups were treated with paclobutrazol (20mL) and same amount of water respectively. The expression levels of the three genes changed significantly during grain development after treatment of exogenous paclobutrazol. Almost all the genes showed a trend of increasing first and then decreasing, and the expression level reached a maximum value at 27D/36D. Compared with the control group, the expression of *SbGRAS68* was lower at 9D, increased significantly at 18D, and reached highest at 27D. Upon paclobutrazol treatment, the expression of *SbGRAS23* increased at 27D, but decreased at 45D. It is worth noting that the expression of *SbGRAS03* increased greatly during the whole grain development stage, and the response was more obvious by the exogenous paclobutrazol treatment.

### Expression patterns of *SbGRAS* genes in response to different abiotic stresses

**Fig. 10:**
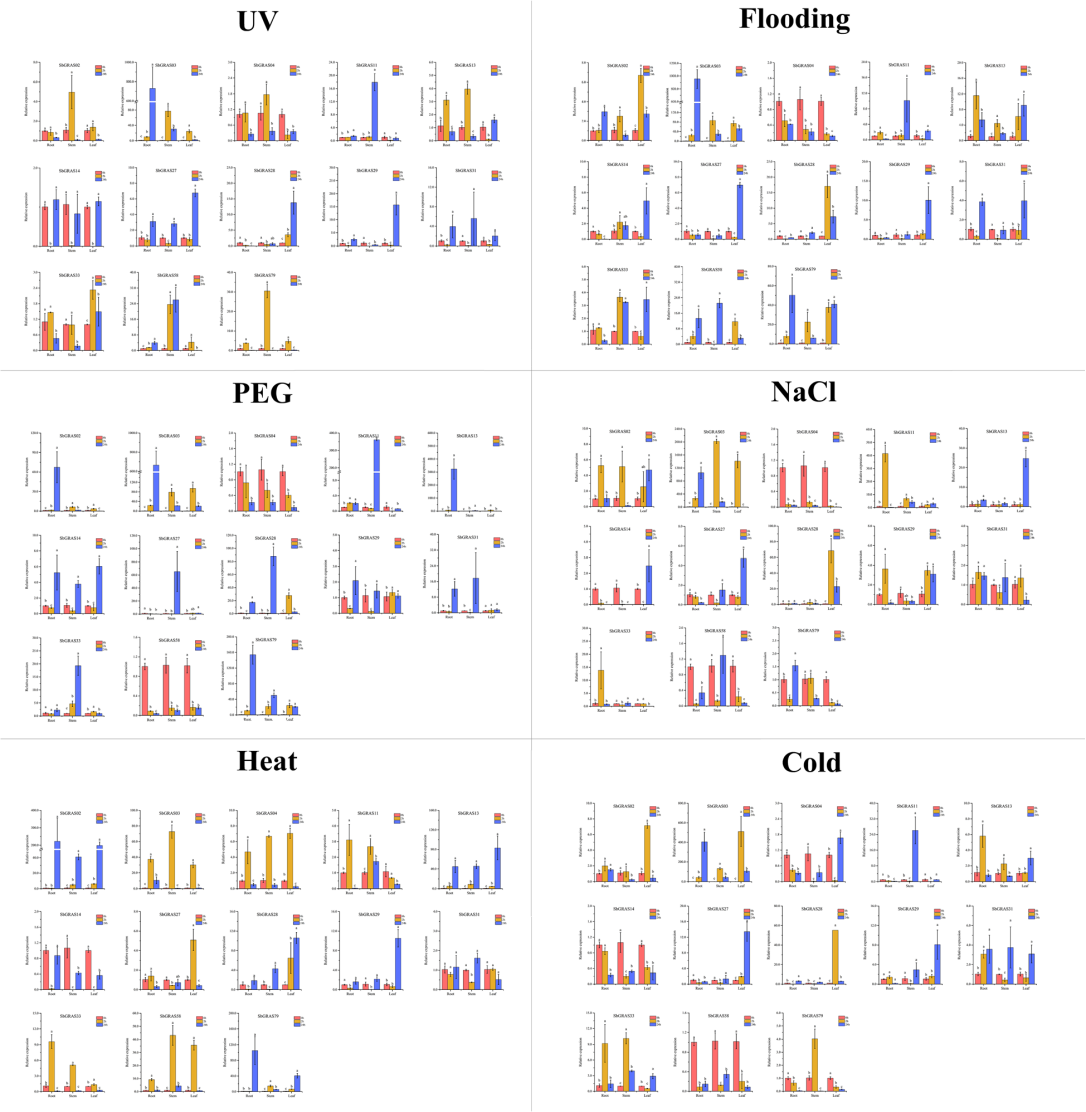
Expression of 13 *S. bicolor GRAS* genes under different abiotic stresses (strong UV radiation, flooding, PEG, NaCl, heat and cold treatments) at the seedling stage. Error bars were obtained from three measurements. Lowercase letters above the bars indicate significant differences (α = 0.05, LSD) among the treatments

To further determine whether the expression of *SbGRAS* genes is influenced by different abiotic stresses, 13 *SbGRAS* members were examined for their expression under six abiotic stresses: strong ultraviolet radiation (UV), flooding, polyethylene glycol (PEG), NaCl, heat and cold treatments. We ran qRT-PCR experiments to analyze the 13 *SbGRAS* members’ expression patterns in roots, leaves and stems in response to the different treatments (Fig. 10). Some *SbGRAS* genes were significantly induced/repressed by a number of the abiotic stress treatments. Expression of most of these genes was significantly altered in the early stage of the treatment (Fig. 10). Among them, some *SbGRAS*s showed changes in expression that were similar or opposite at different times and in different organs. For example, under UV, flooding, PEG, and NaCl treatment, the expression level of *SbGRAS04* decreased significantly in the roots, stems and leaves, indicating its rapid inhibition by these stresses. But its expression was totally opposite under heat and cold stresses: it was initially significantly upregulated and then downregulated in roots, stems and leaves, which expression pattern remained constant. Under all treatments, the expression of *SbGRAS03* (DELLA) in the roots increased significantly, whereas in the stems and leaves, it was initially significantly upregulated and then downregulated. The expression level of *SbGRAS58* increased significantly under UV and flooding treatments, but decreased gradually under PEG treatment.

## Discussion

This study systematically analyzed the *GRAS* gene family in *S. bicolor*, and identified a total of 81 *SbGRAS* genes. All SbGRAS proteins showed significant differences in structure, indicating a high degree of complexity. The lengths of the GRAS proteins varied between 174 and 968 amino acids, indicating great variability [6–8]. This variation may be related to gene-duplication events or genome size [44]. According to the constructed phylogenetic tree (Fig. 1), we identified at least one SbGRAS protein in each subgroup of *A. thaliana*, indicating that the divergence of the GRAS family may have occurred earlier than that of monocotyledons and dicotyledons, with some new subfamily members being produced as evolution proceeded. Among the 13 subfamilies, LISCL had the most members (39, ~ 48.15 %), which is similar to other plants, such as *A. thaliana* [6–8], rice [6–8], and maize [47], indicating that these *GRAS* gene families may have had strong partial differentiation capabilities in the long-term evolutionary process. Most of these genes share five main conserved domains: LHR I, VHIID, LHR II, PFYRE and SAW (Fig. 2). The core VHIID domain contains the characteristic sequence of Val, His, Ile and Asp. The His and Asp residues are more conserved. We divided the HAM subfamily into two groups, HAM-1 and HAM-2, based on their genetic and developmental relationship and the multiple-sequence-alignment results. The N terminus of the SbGRAS proteins contains a highly disordered region, but it shows certain similarities in different subfamilies. For example, the DELLA subfamily protein contains the DELLA domain at its N terminus. This leads to the diversification of GRAS proteins and affects their functional differentiation. We observed that some residues in these regions are absolutely conserved in different subfamilies, and that these residues may be required for GRAS proteins of different subfamilies to function. In general, an IDR in an IDP allows the proteins to recognize and interact with various partners, which is essential for molecular recognization [7, 27, 79].

We analyzed the exon and intron structures of the 81 identified *SbGRAS* genes (Fig. 3, Additional file 1: Table S1); the number of exons per gene ranged from 1 to 5 (Fig. 3A/B). The proportion of *SbGRAS* genes without introns was higher (54, ~ 66.67 %) than in plum blossom (82.2 %), *Arabidopsis* (67.6 %), rice (55 %) and poplar (54.7 %) [68, 80, 81]. Intron-less genes have also been found in other large gene families, such as the DEAD box RNA helicase [82] and F-box TF families, and the small auxin-up RNA (Saur) gene family [83]. Although there are no genes with introns in prokaryotic genomes, one study [47] showed that plant *GRAS* genes originated from the prokaryotic genes mainly by horizontal gene transfer and by duplication events during their evolution. This phenomenon explains the large number of intron-less *GRAS* genes based on sequence homology and classification [8]. Genes without introns are not separated and can therefore be continuously encoded into proteins. in addition, introns are conducive to species evolution: they can increase gene length, increase the frequency of recombination between genes, and they have regulatory effects [84]. Although intron-less genes have no advantage in species evolution or recombination, they tend to respond quickly to stress. Intron-less genes can delay regulatory responses and rapidly regulate the growth and development process [85, 86]. Therefore, many *SbGRAS* members may be able to respond quickly to environmental changes. Ten different conserved motifs were found, and *SbGRAS* members in the same group usually had a similar motif composition (Fig. 3). It is possible that the transcriptional regulators in a taxonomic group have the most recent common evolutionary origin and molecular function, which makes this an effective and practical method to predict the function of unknown proteins [53].

Gene amplification is a very important driving force in genome evolution, which can lead to the emergence of new functional genes and the differentiation of new species, thereby rendering plants more adaptable to harsh environments during evolution [68]. Tandem repeat events occur more frequently in plant genomes, such as *Arabidopsis* and rice, affecting about 10 % of the genes [6]. Compared to *Arabidopsis* and rice, there are more GRAS proteins in sorghum, indicating that there may have been more gene duplication events in sorghum or a higher frequency of retained copies after replication. We found 16 tandem repeat events in the SbGRAS proteins, involving 25 genes (~ 30.9 %), which is higher than in *Arabidopsis* 2/34 [6], plum 10/45 [80], tomato 15/53 [43] or rice 17/60 [6], but lower than in poplar 40/106 [8]. It is worth noting that the two narrow regions of Chr5 contain 4 pairs and 7 pairs, respectively, of closely homologous genes (Fig. 4, belonging to the LISCL subfamily). We found that all *SbGRAS* genes that had tandem repeats came from the same subfamily, and mainly occurred in the LISCL subfamily (14, ~ 87.5 %). This implies that the retention of gene copies is biased to a certain extent after the whole genome is replicated, while the structure does not produce great differences, and the retention and loss of different subgenomes are also different. In addition, some studies have found that if there is an interaction between the protein and other products encoded by the gene, this type of gene will be biased after the replication event has occurred [87]. The contribution of tandem repeats to the increase in sorghum *GRAS* members was slightly greater than that of fragment repeats (23 *SbGRAS* genes). Further analysis of these gene subfamilies showed that they are all linked within subfamilies. These results indicate that some *SbGRAS* genes may have been generated by gene-duplication events, further confirming that duplication events are the most common mechanism leading to the rapid expansion of *GRAS* family members in different species.

Roots, stems, leaves, flowers and grain are the main organs in all angiosperms. Studies have shown that GRAS TFs have participate extensively in the development of flowers and grain in omnipresent plants [88]. *SbGRAS02* showed a higher expression in the flower and grain (Fig. 8A), which was consistent with the expression pattern of the homologous gene AT3G54220. AT3G54220 may play a key role during flowering stage, embryo cotyledonary and globular stage in *Arabidopsis thaliana* [89]. The transcription level of *SbGRAS31* and the homologous gene AT3G60630 were both high in stem, and the AT3G60630 was required for maintenance of shoot in *Arabidopsis thaliana* [90]. *SbGRAS03* of the DELLA family also demonstrated higher expression during the grain-filling stage, suggesting an important role in the development of sorghum grain. *SbGRAS14*, one of the PAT1 members, is highly expressed in grain and involved in PhyA signal transduction [91]. However, specific functions need to be analyzed through in-depth experiments. The expression of most *SbGRAS* genes were significantly positively correlated, suggesting that their combination has a synergistic effect in six plant organs (Fig. 8B). In summary, these results revealed the functions of some GRAS TFs and their self-regulation.

The growth and development of plants will be affected by the external environment and hormones. DELLA protein is an integrator of multiple hormonal signals and environmental signal systems, which regulate growth and development of plants by means of mediating gibberellin [92]. As a central regulator in plant growth and performs, GAs are active through degrading DELLA proteins. Peng [93] proposed that when GAs was absent, DELLA proteins would bind transcription factors, regulated plant growth, development and inhibited downstream genes expression, thereby suppressing plant growth. However, in the presence of GAs, the DELLA proteins were degraded and its inhibitory effects were removed. DELLA plays an important role in the development of plant fruits [53]. For example, in tomato and *Arabidopsis thaliana*, the expression of DELLA genes can induce parthenogenesis [94]. The endogenous gibberellin content was analyzed during the grain development process in *S. bicolor*, and GAs could be detected throughout the whole grain development stage (Fig. 9B), which gradually decreased from 9D (133.18 µg·g^− 1^) to 45D (36.99 µg·g^− 1^). Under mock treatment, the expression of DELLAs, showed an undulating and slow fluctuation during the grain development stage, were relatively stable during the middle stage. Compared with the DELLA subfamily members (*SbGRAS03*, *SbGRAS*23 and *SbGRAS*68), the expression of *SbGRAS*23 during middle grain development was significantly higher than those at early (9D) and late (45D) stages. Therefore, we hypothesized that *SbGRAS23* may play a role during the middle development stages of grain. As a plant growth inhibitor, paclobutrazol regulates plant growth mainly by inhibiting biosynthesis of GAs by regulating DELLAs transcription [53]. In order to further investigate the relationship between DELLAs, GAs and grain development in *S. bicolor*, we sprayed paclobutrazol on germinating plants. The findings show that paclobutrazol treatment (exogenous application of 750 mg·L^− 1^) significantly increased the grain weight of *S. bicolor* (Fig. 9A), particularly in the middle and late stages of grain (27D to 45D). Then, the expression levels of the DELLA subfamily (*SbGRAS03*, *SbGRAS23*, and *SbGRAS68*) in paclobutrazol treated was further analyzed (Fig. 9C). Compared with the mock, the expression pattern of all DELLA genes were significantly changed, especially in the grain from 27D to 36D. The expression level of *SbGRAS68* changed significantly from 9D to 27D, indicating that it may be sensitive in the early stage. The expression level of *SbGRAS23* treated with paclobutrazol showed little difference from that of mock treatment at the early stage (9D ~ 18D), while the expression level increased sharply in the middle stage (27D) and decreased rapidly in the later stage. Interestingly, after the paclobutrazol treatment, the expression level of *SbGRAS03* was remarkably increased during the whole grain development. At the same time, the sensitivity of *SbGRAS03* to polybutrazole treatment was higher than that of *SbGRAS23* and *SbGRAS68*. Therefore, we speculate that *SbGRAS03* may has potential value in sorghum breeding. After treatment with paclobutrazol, the expression levels of the three genes in the DELLA subfamily were significantly different during grain development, which indicated that the functions of the three genes might be different.

We also studied the responses of these 13 typical *SbGRAS* genes to six abiotic stresses in different organs, and found that almost all of them exhibited significant differential expression under stress (more than 2-fold change). For example, under PEG stress, the expression of 9 *GRAS* genes was upregulated in roots, 11 in stems, and 9 in leaves. In this study, *SbGRAS03* was significantly expressed in the root, stem, leaves and grain. At the same time, it was significantly induced under UV, flooding, PEG and NaCl treatments at the seedling stage, and its expression level gradually increased and then fell in the stems and leaves. It reached its highest expression level in 2 h. OsGRAS19 and the brassinosteroid signal-transduction pathway can regulate grain size by promoting cell division and regulating the number of epidermal cells on glumes. Overexpression of the *OsGRAS19* gene or CRISP/Cas9 mutants showed an effect on grain size. *SbGRAS58* had positive effects in the roots, stems and leaves [95], and more significant expression in the grain. What is interesting is that there was also significant expression in the style, suggesting that it may participate in a complex biological development network. Not only that, but *SbGRAS58* exhibited completely different response modes under different stresses. For example, under UV and flooding, expression level in the rhizomes showed a significant gradual upward trend; under heat and PEG stress, the expression level showed a continuous downward trend; under heat stress, the expression level showed a trend of first increasing and then decreasing. We found some differences in the expression patterns of the selected HAM-1 (*SbGRAS31*) and HAM-2 (*SbGRAS13*) genes. We further analyzed their expression in different tissues. The expression of *SbGRAS13* in the grain and style was significantly higher than that of *SbGRAS31*, while in the stem and anther, the opposite was seen. HAM-1 and HAM-2 are two different HAM subfamily members, and *SbGRAS13* and *SbGRAS31* show completely inconsistent expression trends. For example, under UV and flooding stress, *SbGRAS13* first increases and then decreases in roots and stems. The expression trend of *SbGRAS31* first decreased and then increased, indicating that more detailed differentiation may lead to great functional differences in the response to environmental stress. *Arabidopsis* SHR may interact with SCR and SCL23 subfamily proteins to form the SHR–SCR–SCL23 module that regulates root endoderm development [96]. The 7 SHRs and 4 SCRs identified in sorghum may share functions with SHR and SCR in *Arabidopsis*, as their functions in rice and *Arabidopsis* have been shown to be conserved [96]. Nevertheless, like the *Arabidopsis* SHR subfamily, some or all of the loss in gene function of the broader SHR and SCR subfamilies in sorghum needs to be carefully studied in the future, because most of their gene members are expressed at low levels (Fig. 8A and 10). DELLA protein regulates plants’ stress tolerance [97]. In our qRT-PCR results, *SbGRAS03* had an extremely high response under all stress treatments, although the expression levels in different tissues were inconsistent, indicating their important role in coping with adversity. Therefore, *SbGRAS* genes have a potential regulatory role in plant development and responses to stress. These overall findings provide insights into the potential functional roles of sorghum *GRAS* genes, and help understand the developmental process in sorghum toward genetic improvement of environmental resistance.

## Conclusions

In this study, 81 *GRAS* gene family members were identified in the genome of *S. bicolor*, and phylogenetic analysis indicated that these *SbGRAS* genes could be classified into 13 subfamilies. Most of the *SbGRAS* genes were intron-less. It was found that gene-replication events may have produced some *SbGRAS* genes, with tandem duplication contributing more to the expansion of the *SbGRAS* gene family than segmental duplication. Phylogenetic comparison and synteny analysis of *GRAS* genes from six typical plant species provided valuable clues about the evolutionary characteristics of *GRAS* gene family members in *S. bicolor*. The expression patterns of the *GRAS* members of *S. bicolor* under abiotic stresses and in plants exposed to six abiotic stresses at the seedling stage using qRT-PCR. The relationship between DELLA genes, gibberellin content and grain development in *S. bicolor* were further investigated. Paclobutrazol treatment significantly down-regulated gibberellin content and increased grain weight during whole grain development. In addition, *SbGRAS03* in the DELLA subfamily is the most sensitive to the treatment of paclobutrazol, and its expression level is up-regulated in grain development of *S. bicolor*, which was valuable in Sorghum breeding.

## Methods

### Gene identification

We downloaded the *S. bicolor* whole genome sequence information from the Ensembl Genomes website (http://ensemblgenomes.org/). First, with BLASTp (score value ≥ 100 and e-value ≤ 1e − 10), all possible GRAS proteins were identified from the *S. bicolor* genome referring to trihelix protein sequences of *Arabidopsis*. Then, the hidden Markov model (HMM) file corresponding to the GRAS trihelix domain (PF03514) was downloaded from the PFAM protein family database (http://pfam.xfam.org/) [98, 99]. We used both HMMER3.0 (default parameters) with a cutoff of 0.01 (http://plants.ensembl.org/hmmer/index.html) [100] and SMART (http://smart.embl-heidelberg.de/) [101, 102] to ascertain the presence of the GRAS domain. By using the tools from the ExPASy website (https://web.expasy.org/compute_pi/), the 81 *SbGRAS* proteins were obtained the sequence length, molecular weight, pI and subcellular localization.

### GRAS structure

The *SbGRAS* domain sequences of the characterized GRAS proteins were used to create multiple protein sequence alignments using ClustalW with default parameters [103]. The deduced amino acid sequences in the GRAS domains were then adjusted manually using GeneDoc software. To study the structural differences between the *SbGRAS* genes, conserved motifs were studied in the encoded GRAS proteins [104, 105]. We used the gene structure display server (GSDS: http://gsds.cbi.pku.edu.cn) online program to analyze the exon–intron structure of the *SbGRAS* genes based on CDS length and corresponding full-length sequence. An online MEME program (http:/meme.nbcr.net/meme/intro.html) was used to analyze the protein sequences with the following parameters: optimum motif width of 6 ~ 200, and maximum number of motifs, 10 [99, 105, 106].

### Chromosomal distribution and gene duplication

All *SbGRAS* genes were mapped to *S. bicolor* chromosomes based on physical location information from the database of the *S. bicolor* genome using Circos [107]. Analysis of *SbGRAS* gene-replication events was conducted using multiple collinear scanning toolkits (MCScanX) with the default parameters [108]. We analyzed *GRAS* gene homology between *S. bicolor* and six other plants (*A. thaliana*, *C. annuum*, *Solanum lycopersicum*, *B. distachyon*, *O. sativa* subsp. *indica*, *Z. mays*) using Dual Synteny Plotter (https://github.com/CJ-Chen/TBtools).

### Phylogenetic analysis and classification of *SbGRAS* gene family

According to the classification of the *AtGRAS*s, the identified *SbGRAS* genes were divided into different groups. The phylogenetic trees were inferred using the NJ method of MEGA X via Geneious R11 with the BLOSUM62 cost matrix, the Jukes–Cantor model, global alignment with free end gaps and bootstrap value of 1000. The full-length amino acid sequences of the GRAS proteins (Additional file 1: Table S1) derived from *(A) thaliana*, *C. annuum*, *Solanum lycopersicum*, *(B) distachyon*, *O. sativa* subsp. *indica*, and *Z. mays* (UniProthttps://www.uniprot.org/) combined with the newly identified SbGRASs were used for phylogenetic analysis.

### Plant materials, growth conditions, paclobutrazol and abiotic stress in *S. bicolor*.

The *S. bicolor* variety Hongyingzi materials used in the experiment were supplied by Prof. Cheng Jianping of Guizhou University. ‘Hongyingzi’ has been grown in the greenhouse at Guizhou University since 2019. *S. bicolor* plants were grown in pots filled with soil and vermiculite (1:1) in a growth room with a 16 h/25℃ day and 8 h/20◦C night regime, and a relative humidity of 75 %. We collected the stems, roots, leaves, grain, anthers and styles separately from five plants with good growth and similar growth conditions, and quickly placed them in liquid nitrogen for storage at − 80 °C for further use. Hongyingzi materials with similar growth statuses were selected and sprayed with 20 mL paclobutrazol (750 mg·L-1) during the germination period. The same amount of water was sprayed as a blank control. Grain samples were collected at 9D, 18D, 27D, 36D, 45D, respectively. Several *SbGRAS* genes were selected to investigate their expression patterns in response to various stresses. *S. bicolor* plants at the seedling stage (21 days) were selected for the abiotic stress treatments, which included salt treatment (5 % NaCl), water flooding (whole plant), drought (30 % PEG6000), UV radiation (70 µW/cm^2^, 220 V, 30 W), high temperature (40 ℃), and low temperature (4 ℃). Each stress treatment had five replicates; qPCR detection and hormone (GA) analysis were carried out after sampling at 2 and 24 h, respectively. The collected samples were stored at − 80 °C for subsequent analysis.

### Total RNA extraction, cDNA reverse transcription and qRT-PCR analysis

The cDNA was produced with a 1 mg RNA sample using a PrimeScript RT Reagent Kit with gDNA Eraser (TaKaRa) and SYBR Premix Ex Taq II (TaKaRa) [100]. The sequencing was performed in an Illumina GAII sequencer following the manufacturer’s instructions [105, 106]. Gene-expression analysis of the selected genes was performed by qRT-PCR, and repeated at least three times, with primers designed by Primer 5.0 (Additional file 8: Table S8). We used the *GAPDH* (glyceraldehyde-3-phosphate dehydrogenase) gene, which was stably expressed at each growth stage in almost all tissues, as the internal control [109]. The correlations of expression data were calculated according to the 2^−(ΔΔCT)^ method [110].

### Endogenous GA analysis

We added 20 mL of 80 % ethanol to 1 g of fresh sample. Then the samples were extracted three times with ultrasound, for 1 h each time. The extract was concentrated once at low temperature and mixed with water, and then extracted with an equal volume of N-butanol; the N-butanol layer was dried under a nitrogen stream. Then, 20 mg of the dried sample was dissolved in 5 mL methanol (MS grade), and filtered through a 0.22-µm membrane. The chromatographic conditions were: a C18 column (2.1 mm × 75 mm, 2.7 μm) held at a constant 40 °C; injection volume 5 µL; the mobile phase was acetonitrile (A) and deionized water (B); the mobile phase gradient elution was 0 min ~ 1 min, 20 % A; 1 min ~ 1.5 min, 80 % A; 1.5 min ~ 4.5 min, 80 % A; 4.5 min ~ 6 min, 20 % A; 6 min ~ 8 min, 20 % A.

### Statistical analysis

Analysis of variance (ANOVA) was performed with JMP6.0 software (SAS Institute), and compared by least significant difference (LSD) at the 0.05 and 0.01 levels. The histogram was drawn with Origin 8.0 software (SAS Institute).

## Supplementary Information

**Additional file 1: Table S1.** List of the 81 *S. bicolor GRAS*genes identified in this study.

**Additional file 2: Table S2.** Subfamilies and protein sequences of *Arabidopsis* and rice.

**Additional file 3: Table S3.** Analysis and distribution of the conserved motifs of GRAS proteins.

**Additional file 4: Table S4.** Tandem duplication events of *S. bicolor GRAS* genes.

**Additional file 5: Table S5.** The 14 pairs of segmental duplicates in *S. bicolorGRAS* genes.

**Additional file 6: Table S6.** One-to-one orthologous relationships between *S. bicolor* and other plants.

**Additional file 7: Table S7.** Results of Tajima's D neutrality test.

**Additional file 8: Table S8.** Codon-based Test of Positive Selection for analysis between sequences.

**Additional file 9: Table S9.** Primer sequences for qRT-PCR.

## Data Availability

The *Sorghum bicolor* whole genome sequence information is from the Ensembl Genomes website (http://ensemblgenomes.org/). The *Sorghum bicolor* materials (Hongyingzi) used in this study were supplied by Prof. Cheng Jianping of Guizhou University. The datasets supporting the conclusions of this article are included in the article and its Additional files.

## Abbreviations

At: *Arabidopsis thaliana*
Os: *Oryza sativa*
AtGRAS: *Arabidopsis thaliana* GRAS
OsGRAS: *Oryza sativa* GRAS
SbGRAS: *Sorghum bicolor* GRAS
GAPDH: Glyceraldehyde-3-phosphate dehydrogenase
qRT-PCR: Quantitative real-time polymerase chain reaction
TF: Transcription factor
CDS: Coding sequence
HMM: Hidden Markov Model
pI: Isoelectric point
BLAST: Basic local alignment search tool
LHR I: Leucine-heptad repeat I
LHR II: Leucine-heptad repeat II
SCR: SCARECROW
